# 

**Published:** 1892-03-19

**Authors:** 


PRICE ONE PENNY.

No. 286, Vol. XI.—March 19th, 1892.

[Registered at the General Post Office as a Newspaper for transmission in the United Kingdom and Abroad.]

WITH EXTRA NURSING SUPPLEMENT.
Per Annum, 6s. 6d., post free.
Monthly Parts, 6d.; post free, 8d.
Ditto per Annum, 8s., post free.

### THE HOSPITAL

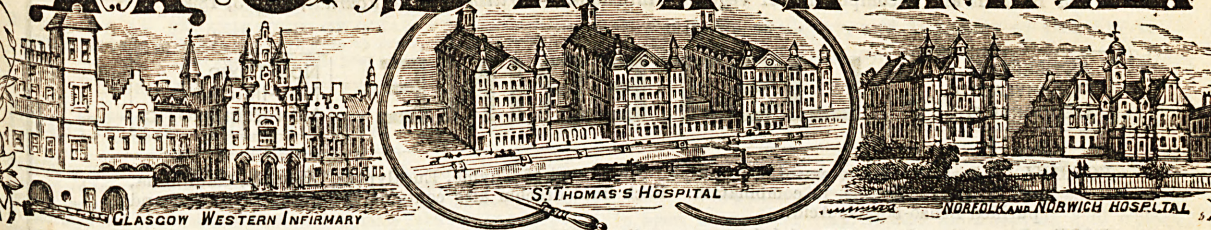

A WEEKLY JOURNAL OF

### Science, Medicine, Nursing, and Philanthropy.

SATURDAY, MARCH 19th, 1892.

**Some Conditions of Medical Progress** - 297

**Passing Topics.**—The Festival Season - 298

**The Doctor's Armchair.**—Hypnoti m in America - 299

**Letters from Far Japan.**—By Mrs. Ernest Hart. III.—Medical Banquets - 300

**Some Scientific Aspects of Insanity.** XVII.—The Insanities of the Puerperal State - 301

**Everybody's Page** - 301

**Notes and News** - 302

**Annotations.**—Neurotic Novelists—The Bloodlessness of School Girls—The Miners' Holiday - 304

**General Charities—Scraps and Gleanings** - 3.3

**The Practitioner's Mirror and Retrospect—**

Medicine.—The Therapeutics of Oxygen Gas - 305

Therapeutics.—Salophene—Some Uses of Antimony - 306

New Drugs, Appliances, and Things Medical.—Lanura Flannel—Braided Wire Cushions and Seats—Sacklin's Patent Poultices—Blankets - 307

**Talks About Food.** IV.—Sucking the Honey from the Bee - 308

**Turning Over New Leaves** - 308

**The Student of Medicine.**—Appointments—Vacancies—Notes from the Schools—Pass Lists—Athletics.

EXTRA SUPPLEMENT.—THE NURSING MIRROR.

**En Passant.**—Nurses' Cases—St. Patrick's Home—A Residential Club—Nursing Guilds—Bolton Infirmary—Short Items—District Mursing - cxlv

**On the Nursing of Children.** VI.—Splints (*continued*) cxlvi

**Medical Opinion on Registration** - cxlvi

**Wants and Workers** - cxlvi

**Nursing Uniforms.** III.—Russian Nurses - cxlvii

**Notes and Queries** - cxlvii

**For Reading to the Sick**—"Wings" - cxlvii

**Nurses for the Insane.** By a Medical Officer - cxlviii

**Where to Go** - cxlviii

**Everybody's Opinion**—District Nursing Disagreements - cxlix

**To a Nurse** - cxlix

**Death in Our Ranks** - cxlix

**Appointments** - cxlix

**Off Duty.**—Ugliness *versus* Beauty - cl

